# Locked down: Orthopaedic surgery in a regional health service during the COVID‐19 pandemic

**DOI:** 10.1111/ans.70007

**Published:** 2025-02-03

**Authors:** Thomas Page, Margaret Rogers, Kevin Eng, Kirsten Porter, Stephen D. Gill, Richard S. Page

**Affiliations:** ^1^ Orthopaedic Department of University Hospital Geelong Barwon Health Geelong Victoria Australia; ^2^ Surgical & Critical Care Directorate Barwon Health Geelong Victoria Australia; ^3^ Barwon Centre for Orthopaedic Research and Education (BCORE) Deakin University, St John of God Hospital Geelong and Barwon Health Geelong Victoria Australia

**Keywords:** arthroplasty, COVID‐19, orthopedic procedures, orthopedics, replacement, waiting lists

## Abstract

**Background:**

COVID‐19 affected access to healthcare. Victoria, Australia was heavily affected. Hospitals deferred non‐urgent operations and preserved health resources to manage people with COVID‐19. Elective orthopaedic surgery was directly impacted. This study investigated changes to orthopaedic procedures and the elective surgery waitlist during the COVID‐19 at a large regional health service in Victoria.

**Methods:**

Data were acquired from University Hospital Geelong, a publicly funded regional health service in Victoria, Australia. Orthopaedic surgeries and waitlist numbers were collated for financial years ending (FYE) 2020–2023. Procedures were displayed as total, planned and unplanned (i.e., trauma).

**Results:**

From FYE 2020 to 2023 there was 8244 orthopaedic surgery cases with 8850 procedures. Planned joint replacements of knee, hip and shoulder decreased collectively by 19% in FYE 2022 and increased by 66% in FYE 2023. Waiting lists rose from 247 in FYE 2020 and peaked at 786 in FYE 2022, before falling to 390 in FYE 2023. The number of fractured neck of femur procedures were consistent each year (average 152/year), while ankle and wrist fractures declined by 55% and 43% during FYE 2022 and increased in FYE 2023 by 106% and 159% respectively.

**Conclusion:**

Changes to planned orthopaedic procedures and waitlists in a regional centre were evident throughout the pandemic. These results can help to inform strategies to optimize the provision of orthopaedic surgery during future major disruptive events.

## Graphical abstract

COVID‐19 affected access to healthcare, and orthopaedic surgery was significantly impacted. Wrist and ankle fractures declined, whilst neck of femur fractures were constant throughout the pandemic. Waiting lists increased after limitations were imposed on surgical activity, resulting in decreased planned orthopaedic surgery. Waiting lists then declined following enhanced operating capacity.

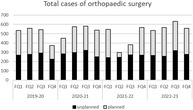

## Introduction

Orthopaedic services are critical in the management of musculoskeletal disease which is one of the biggest contributors to global disability, most commonly as osteoarthritis (OA).^1^ OA reduces quality of life, increases comorbidities, and inflicts significant healthcare costs. Advanced OA can be cost‐effectively managed with joint replacement surgery.^2^ In 2019, immediately prior to the COVID‐19 pandemic, 125 627 joint replacements were completed in Australia.^3^ Additionally, fractures following trauma are common, accounting for approximately one fifth of emergency presentations.^4^

Healthcare systems globally were challenged during the COVID‐19 pandemic. Nearly 1 month following the first recorded COVID‐19 case in Wuhan China, Australia reported its first case on 25th January 2020. In Victoria, the impact of successive lockdowns (legal limitations on leaving home) and the associated cessation of elective or planned surgery had a significant impact on healthcare. Concurrently, surgery was restricted to urgent cases from March 26th 2020 until mid‐2022.^5^ Regional public hospitals in Victoria held the unique position of following less stringent regional restrictions with broader travel limits and greater community access. There was also an increase in regional population size and subsequent healthcare burden due to those ‘escaping’ Melbourne, numbering over 9000 in financial year ending (FYE) 2020 alone.^6^

Restricting planned surgery was a strategy to maintain the availability of ward and ICU beds for people with COVID‐19, and to ensure sufficient supply of protective equipment could be maintained. Consequently, surgical resources were primarily directed to managing trauma and urgent planned (Category 1 and select Category 2) cases only. Surgical staff were re‐allocated to other areas of need within health services. This redirection of priorities and changes to orthopaedic presentations, such as trauma in the lockdown environment, drove new trends in the types of orthopaedic procedures completed in regional Victoria and similarly abroad.^7^, ^8^ Non‐urgent planned waitlists grew rapidly throughout these years creating a backlog of elective orthopaedic procedures and outpatient appointments.

The aim of this study is to report the changes in orthopaedic presentations and procedures performed in a regional Victorian public health service from FYE 2020 to FYE 2023 The reduction in surgical access, impact on waitlists and subsequent ‘catch up’ strategies are explored. The study reflects upon a unique period of altered orthopaedic activity and identifies key learnings that can be used to help optimize management of orthopaedic conditions both now and for future disruptive events.

## Methods

University Hospital Geelong is a publicly funded hospital that provides surgical services for the Greater Geelong and southwestern Victoria region. All acute orthopaedic services, excluding complex spinal or significant multi‐trauma are provided, alongside the full spectrum of joint replacement surgery.

Data were acquired from the Business Management Data Warehouse of the regional Victorian health service of University Hospital Geelong within Barwon Health, for the COVID‐19 pandemic period. Total cases of orthopaedic surgery have been collated for the FYE 2020, 2021, 2022 and 2023 based upon surgical coding numbers. The 2020 financial year began on July 1st 2019 and ended on June 30th 2020.

The provision of health services at University Hospital Geelong were affected by the following regional lockdown dates:

- 31st March–12th May 2020
- 9th July 2020–16th September 2020
- 13–17th Feb 2021
- 28th May–3rd June 2021
- 16–27th July 2021
- 5th–9th August 2021
- 21st August–9th September 2021
- 20th September–22nd October 2021

During periods of stay‐at‐home orders, only five reasons to leave home were permissible: essential shopping, limited caregiving, authorized work or study, vaccination, and exercise within 5 km (later 10 km) from home residence.^9^, ^10^

Waiting list numbers over the financial year quarters (FQ1, FQ2, FQ3, and FQ4) are presented. Procedures are displayed as total procedures, planned (elective joint replacement) and unplanned (acute trauma). Trauma activity was measured by tracking the most common trauma surgeries in our unit, these included distal radius fractures, ankle fractures, and neck of femur fractures (NOF). These procedures were thought to be most representative of the total orthopaedic workload. The number of surgeries for the two pre‐pandemic quartiles (FQ1 and FQ2 FYE 2020) were projected as the expected values post‐pandemic and compared to the exact values using a one sample Chi‐square statistic to determine p‐values. This study was approved by Barwon Health's Human Research and Ethics Committee (Project QA/105690/VICBH‐2024‐413 680(v4)).

## Results

**Fig. 1:**
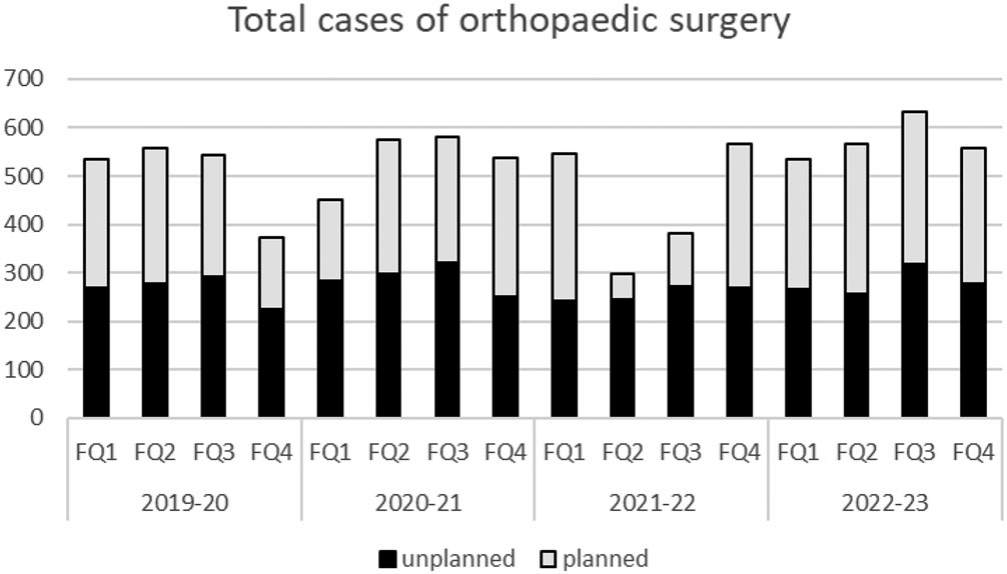
Orthopaedic surgery cases over the pandemic years including FYE 2020 to 2023. Australia's first pandemic case was in January 2020 (i.e., FQ3 FYE 2020).

For the FYE 2020, 2021, 2022 and 2023, there were 2008, 2148, 1793 and 2295 total cases of orthopaedic surgery, respectively (Fig. 1) and 55% of total cases were unplanned. Planned surgery was more heavily affected than unplanned cases. It is evident from Figure 1 that a decrease in total orthopaedic procedures – driven by less planned cases – occurred during select lockdown periods. The FQ1 and FQ2 for the FYE 2020 are pre‐pandemic. Comparing these two periods across the graph the differences are lower for: FQ4 FYE 2020, FQ1 FYE 2021 and FQ2‐3 for FYE 2022 (*P* < 0.05).

**Fig. 2:**
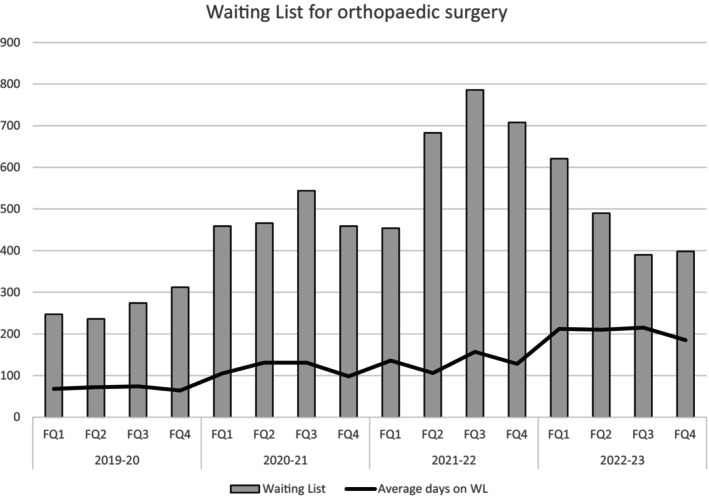
Waiting List for orthopaedic surgery over the pandemic period. Australia's first pandemic case was in January 2020 (i.e., FQ3 FYE 2020).

After FYE 2020 there was an increase in the elective surgery waitlist following lockdown periods (Fig. 2). FQ1 waitlist numbers were 247 in FYE 2020, rising to 459 in 2021, 454 in 2022 and 621 in 2023. FQ2 waitlist numbers from FYE 2020–2023 were 236, 466, 683 and 490, respectively. FQ3 waitlist numbers rose from 274 in FYE 2020 to 544 in 2021 and 786 in 2022 before decreasing to 390 in 2023. Similarly, FQ4 waiting list rose from 312 in FYE 2020 to 459 in 2021 and 708 in 2022 before decreasing to 398 respectively in 2023. The waitlist peaked in FQ3 of 2022 at 786 following the final FQ2 lockdown, equating to a 187% increase from FQ3 2020 (*n* = 274). Hereafter, waitlist numbers reduced by approximately 100 patients each FQ, reaching 390 in FQ3 of 2023, a 50% decrease from the FQ3 2022 peak. Notably, this decrease remained 65% higher than pre‐pandemic waitlist numbers in FQ2 2020 (236).

**Fig. 3:**
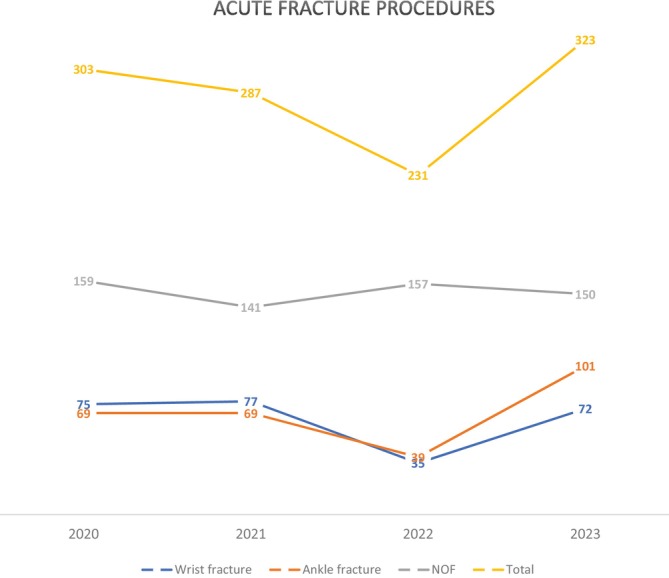
Common acute trauma fractures (unplanned procedures) operated on throughout the pandemic, including wrist, ankle and neck of femur (NOF); by financial year (i.e., 2020 = FYE 2020).

Total common acute unplanned procedures – distal radius, ankle and NOF fractures – declined from 303 in FYE 2020, to 287 and 231 in 2021 and 2022 respectively. FYE 2022 saw a 53%–55% and 43% decline in the number of wrist and ankle fractures respectively compared to 2021 and 2020. FYE 2023 saw an increase in acute fracture operations to 323 up 40% from FYE 2022 (Fig. 3). This was driven by a greater number of wrist and ankle fractures increasing 106% (*n* = 72) and 159% (*n* = 101) respectively. The number of NOF fracture procedures were consistent throughout the pandemic averaging 152 (maximum 159 FYE 2020 and minimum 141 FYE 2021).

**Fig. 4:**
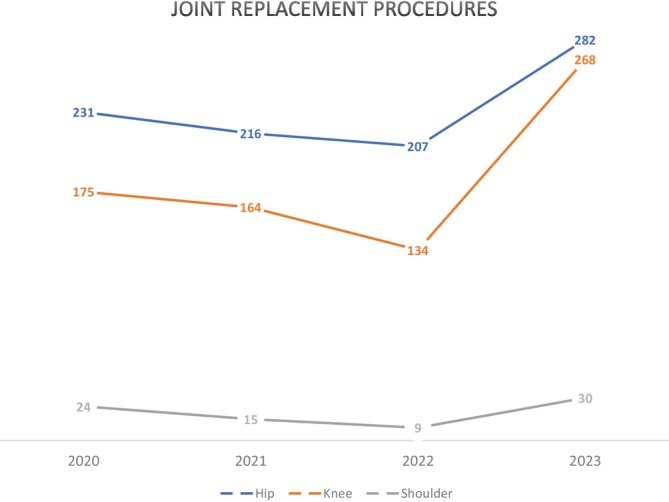
Hip, knee and shoulder joint replacements (planned procedures) by financial year (i.e., 2020 = FYE 2020).

The pandemic had a marked impact on the number of joint replacements performed (Fig. 4). Hip, knee and shoulder replacements declined from 231, 175 and 24 procedures respectively in FYE 2020 down to 216, 164 and 15 procedures in 2021, before falling to 207, 134 and 9 procedures, respectively, in 2022. This represents a 10%, 23% and 63% decrease in hip, knee and shoulder replacements respectively from 2020 to 2022, and a 19% decrease collectively. All procedures increased markedly in FYE 2023 with 282 hip, 268 knee and 30 shoulder replacements performed – representing a 36%, 100% and 233% increase respectively, a 66% increase collectively. This equates to a 35% increase in planned orthopaedic surgery in FYE 2023 compared to the average number (*n* = 1055) of planned procedures across the preceding 3 years.

## Discussion

The effects of the COVID‐19 pandemic were profound at our health service. Total planned orthopaedic procedures declined as seen during financial year quartiles FQ4 2020, FQ1 2021, and FQ2–3 2022 (Fig. 1). Subsequently, waitlists rose following periods of reduced surgical activity. This led to an increased average days on waitlists.

There was then a rebound increase in all procedures in FYE 2023 when limitations were removed and surgical capacity reinstated. Planned hip, knee and shoulder joint replacements had decreased by 19% in FYE 2022 and increased by 66% in FYE 2023. Unplanned surgery for ankle and wrist fractures declined by 49% during lock down periods and afterwards increased by 233%. Fractured NOF procedures were consistent throughout the pandemic. Waitlists rose 186% after lockdown restrictions ceased on non‐urgent procedures (Fig. 2) and peaked in FYE 2022 (*n* = 786), before falling in FYE 2023 (*n* = 390).

Timely access to healthcare is a central component of high‐quality healthcare and includes the provision of elective procedures such as joint replacement surgery. As expected in our study, the size of waitlists was inversely related to the volume of elective procedures being conducted at different times during the pandemic. Waitlists increased following lockdown periods and declined later in the study period when planned procedures were fully re‐instated. These findings align with Victoria's total elective surgery waitlist which swelled to over 80 000 by the end of 2021, a 60% increase on 2019, and to over 90 000 early in 2022.^11^ Across a similar period, the average number of days that a patient was on a waitlist prior to surgery nearly doubled.^11^

The impact of the pandemic on orthopaedic surgery were more pronounced for planned procedures. The low numbers of joint replacements in FYE 2022 relate directly to lockdown periods across 2021 and ongoing surgical limitations into the first half of 2022. Similar to our findings, major reductions in numbers of both total and elective orthopaedic procedures were seen internationally including in the USA, Europe, and the UK during the pandemic.^8^, ^12^, ^13^

Wrist and ankle fractures declined throughout the lockdown years FYE 2020–2022. This is presumed to be due to reduction in physical activities leading to trauma related injuries, for example, sports injuries, motor vehicle accidents (MVA), or falls outside the home.^14^, ^15^ By contrast, there are some findings of increased trauma procedures in Italy at designated trauma centres.^16^ NOF fractures are fragility fractures^17^ and less commonly related to high energy trauma.^18^ NOF fractures through this period were more constant at approximately 150 per year. One study has found similar findings for hip fractures^18^ while another recorded a decrease over the pandemic.^19^

At our regional health service, various strategies were implemented when elective activity resumed to address elevated waitlist numbers. These included:

- Conducting planned surgical activity for public patients in private hospitals.
- Transferring ambulant trauma patients to private hospitals.
- Optimizing theatre occupancy by covering surgeons leave internally or employing locums so theatre space was utilized as efficiently as possible.
- Weekend planned theatre lists were Government funded.
- Implementation of preadmission clinics and short stay orthopaedic surgery enhanced recovery after surgery (ERAS) protocols^20^

Implementing ERAS protocols has been shown to be safe and effective in reducing there length of stay allowing for increased surgical throughput.^20^ In a Victorian study, Christelis *et al*. noted using an ERAS program reduced length of stay and increased the proportion of patients ready for discharge on day 3 following the hip and knee arthroplasty.^21^

Limitations of the current study were identified. This study was conducted with data from one public regional centre in Victoria from FYE 2020 to 2023. The study does not encompass or allow for comparison of the effects the pandemic upon orthopaedic activity in the private setting where 70% of joint replacements occur,^22^ to metropolitan centres, or other regional centres. We acknowledge that any perceived associations between lockdown timing and surgical waitlists is observational and potential confounders have not been assessed. We cannot definitively say whether the increased throughput of planned surgeries and reduction in waitlist were due to these strategies, reduction in lockdowns or a combination of both. However, changes in surgical activity are aligned with lockdown periods and are consistent with other studies.^8^, ^12^, ^13^, ^18^ We hope these findings help inform future management strategies in optimizing timely treatment of patients.

It is challenging to interpret the impact of lockdown versus hospital policy and strategies on operative volume. However, our waiting list rose following periods of lockdown and fell after we implemented the strategies listed above. We hope to integrate these into our routine care models to optimize patient flow.

The COVID‐19 pandemic imposed unique challenges worldwide and had a significant impact on surgical activity, including in regional Victoria as resources were preserved for hospitalized patients with COVID‐19. Important learnings can be identified from the current study in preparedness for future pandemics. Strategies that may be useful ongoing include optimizing theatre time in public and private hospitals, early transfer of ambulant trauma to private, ERAS protocols to increase surgical throughput, and instilling technology to facilitate more efficient outpatient activity. This study demonstrates a reduction in traumatic fracture procedures and reduced number of elective procedures undertaken, whilst procedure numbers for fragility fractures remained stable.

## Author contributions

**Thomas Page:** Conceptualization; investigation; writing – original draft; writing – review and editing. **Margaret Rogers:** Conceptualization; data curation; formal analysis; methodology; supervision; writing – review and editing. **Kevin Eng:** Conceptualization; supervision; writing – review and editing. **Kirsten Porter:** Validation; writing – review and editing. **Stephen D. Gill:** Writing – review and editing. **Richard S. Page:** Conceptualization; project administration; supervision; validation; writing – review and editing.

## Conflict of interest

Prof. Richard Page is an Editorial Board member of Health Science Reports and a co‐author of this article. To minimize bias, they were excluded from all editorial decision‐making related to the acceptance of this article for publication. There are no other conflicts of interest, financial supports, or relationships to declare alongside this submission.
